# Food environment in Brazil: food deserts and food swamps in large urban cities

**DOI:** 10.11606/s1518-8787.2026060007379

**Published:** 2026-07-17

**Authors:** Giovani William Gianetti, Mariana Giannotti, Gabriel Caldeira, Naila Takahashi, Cláudia Roberta Bocca Santos, Ariene Silva do Carmo, Rodrigo Fernando Maule, Gisele Ane Bortolini, Ronaldo Torres, Rogério de S Nóia-Júnior, Bruna Pitasi Arguelhes, Patrícia Chaves Gentil, Lilian Rahal dos Santos, Márcia Muchagata, Sergio Paganini Martins

**Affiliations:** I Universidade de São Paulo. Instituto para Governança Territorial e Políticas Públicas. Piracicaba, SP, Brasil; II Universidade de São Paulo. Escola Politécnica. São Paulo, SP, Brasil; III Centro de Estudos da Metrópole. São Paulo, SP, Brasil; IV Ministério do Desenvolvimento e Assistência Social, Família e Combate à Fome. Secretaria Nacional de Segurança Alimentar e Nutricional. Brasília, DF, Brasil; V Universidade Federal de Ouro Preto. Departamento de Nutrição Clínica e Social. Ouro Preto, MG, Brasil

**Keywords:** Food Environment, Public Policy, Food Security, Public Health

## Abstract

**OBJECTIVE:**

To assess the availability of healthy and unhealthy food establishments in Brazil and, thereby, map food deserts and food swamps. To analyze maps of food deserts and food swamps in conjunction with indicators of social inequality.

**METHODS:**

Data from the Relação Anual de Informações Sociais (RAIS, 2022), integrated with consumption profiles from the Pesquisa de Orçamentos Familiares (POF, 2017–2018), were used to classify establishments according to the Dietary Guidelines for the Brazilian Population. At the municipal level, the density of establishments with healthy and unhealthy profiles was estimated for Brazilian municipalities. At the intramunicipal level, food deserts and food swamps were identified in urban municipalities with more than 300,000 inhabitants, by cross-referencing the results with income indicators from the Cadastro Único.

**RESULTS:**

At the municipal level, it was observed that the North and Northeast regions concentrate most municipalities with low densities of healthy establishments, while the South and Southeast have higher densities of unhealthy establishments. At the intramunicipal level, approximately 25 million people reside in food deserts, corresponding to 32.3% of the population of the 91 municipalities analyzed, and approximately 15 million live in food swamps (19% of the population). Among individuals in the Cadastro Único with a *per capita* income below half the minimum wage, 38% live in food deserts and 10% in food swamps.

**CONCLUSION:**

The results highlight the overlap between socioeconomic vulnerability and adverse food environments, reinforcing the urgency of public policies aimed at regulating the food environment, strengthening public food supply infrastructure, and promoting territorial equity in access to healthy foods.

## INTRODUCTION

Brazil is undergoing rapid social, economic, and territorial transformations, marked by advancing urbanization, the intensification of extreme weather events, and the persistence of structural inequalities that limit access to adequate and healthy food^1^. In 2022, severe food insecurity affected 33 million people, or 15% of the population, 27 million of whom lived in urban areas^2^. In 2023, this number fell to 8.7 million people, of whom more than 7 million lived in cities, reflecting advances in social protection policies^3^. By 2024, 6.48 million Brazilians were experiencing hunger. Despite this, multiple forms of malnutrition persist, including micronutrient deficiencies, overweight, and obesity. Between 2006 and 2023, the prevalence of overweight adults rose from 42.6% to 61.4%, and obesity more than doubled, reaching 24.3%^4^.

This scenario reflects an accelerated dietary transition, marked by the replacement of fresh or minimally processed foods with ultra-processed foods, particularly among socially vulnerable groups. Estimates indicate that these products already account for 19.7% of national caloric intake, with more pronounced growth in rural areas, among black and indigenous populations, those with lower levels of education and income, and in the North and Northeast regions^1^. This dynamic is sustained by hegemonic food systems, organized according to the logic of industrialization and standardization, to the detriment of local and sustainable practices. Such systems are recognized as drivers of the so-called global syndemic, characterized by the convergence of malnutrition, obesity, and climate change, which share common determinants and reinforce one another, amplifying risks for the most vulnerable populations^5^.

One of the central components of these systems is the food environment, defined as the set of physical, economic, political, and sociocultural factors that shape food choices and directly influence consumption and health patterns^8^. In this context, the concepts of food deserts and food swamps have been widely employed. Food deserts correspond to areas with limited availability and accessibility of healthy foods, while food swamps are characterized by the predominance of establishments that primarily sell ultra-processed foods^9^. Both represent spatial expressions of inequality in access to adequate diets and are associated with poorer health indicators^10^.

Mapping these environments is strategically important for guiding public policies on food and nutritional security (FNS). In 2018, the Câmara Interministerial de Segurança Alimentar e Nutricional (CAISAN – Interministerial Chamber for Food and Nutritional Security) conducted the first national survey of food deserts. More recently, the Ministério do Desenvolvimento e Assistência Social, Família e Combate à Fome (Ministry of Development, Social Assistance, Family, and the Fight Against Hunger) committed to updating this mapping when it presented a new strategy focused on the urban food environment.

In this context, the present study aims to assess the availability of healthy and unhealthy food establishments in all Brazilian municipalities and to map the presence of food deserts and food swamps in municipalities with more than 300,000 inhabitants, integrating data on social inequality. The study seeks to provide a comprehensive and comparable view of the urban food environment, informing regulatory and territorial planning strategies that expand equitable access to healthy foods.

## METHODS

This is an ecological study that characterized the density of commercial food establishments at the national level (n = 5,570), using Brazilian municipalities as the unit of analysis; and the mapping of food deserts and food swamps at the intramunicipal scale for all Brazilian municipalities with more than 300,000 inhabitants (n = 91), using hexagons within each municipality as the unit of analysis.

**Figure 1:**
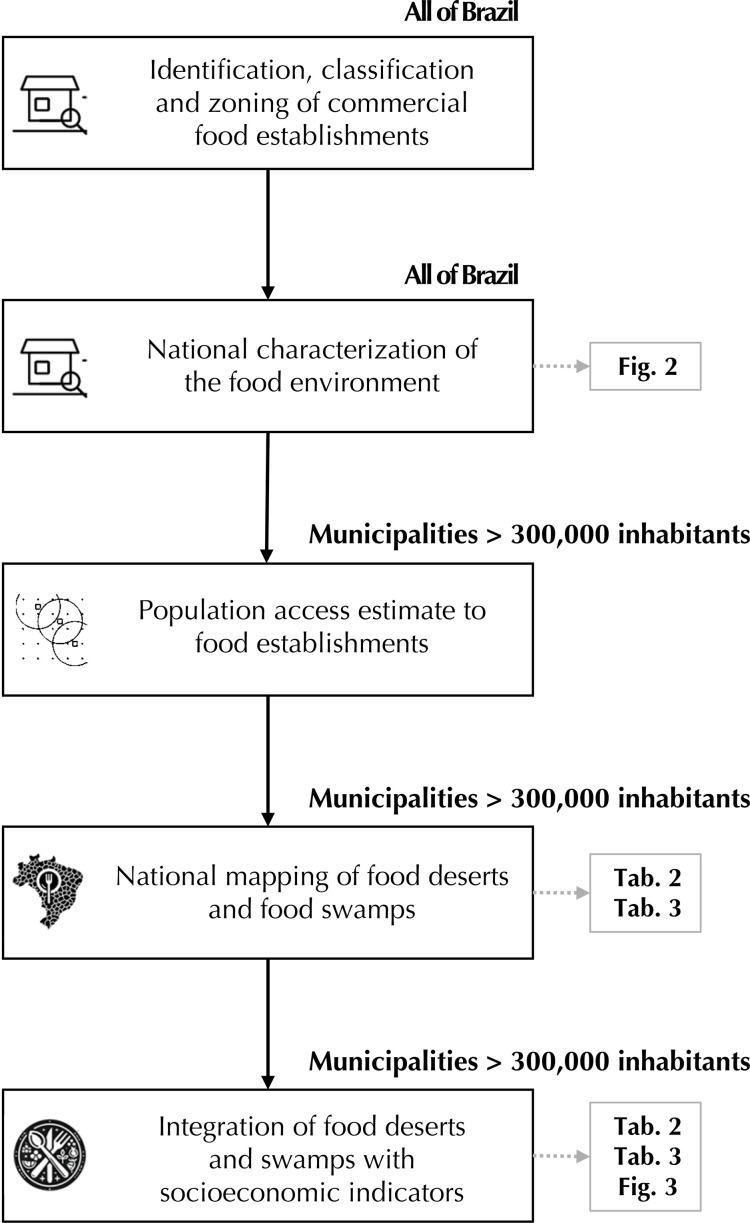
Methodological flowchart for the construction of the typology and mapping of food environments in Brazil.

The study was structured into four integrated stages (Figure 1):

- Step 1 – Identification, classification, and zoning of food retail establishments.
- Step 2 – Characterization of the food environment at the national level: we analyzed all 5,570 Brazilian municipalities to characterize the availability of food establishments classified as healthy (predominantly offering un, fresh, or minimally processed foods) and unhealthy (predominantly offering ultra-processed foods). The metric used was the density of establishments per 10,000 inhabitants. To highlight inequalities in distribution, the 25th percentile (P25) of the density of healthy establishments and the 75th percentile (P75) of the density of unhealthy establishments were considered.
- Steps 3 to 5 – Analysis at the intramunicipal level. The analysis focused on the 91 Brazilian municipalities with a population exceeding 300,000 inhabitants: in Step 3, population access to fresh foods was estimated using density and spatial distribution metrics; in Step 4, food deserts (scarcity of healthy establishments) and food swamps (predominance of unhealthy establishments) were mapped; and in Step 5, the results were linked to socioeconomic indicators, revealing the overlap between social inequalities and restrictions on access to healthy foods (Figure 1).

### Identification, Classification, and Zoning of Establishments

Food retail establishments were identified using the database from the Relação Anual de Informações Sociais (RAIS – Annual Social Information Report) for the year 2022^10^, utilizing the 18 codes from the Classificação Nacional de Atividades Econômicas (CNAE – National Classification of Economic Activities) that cover food purchase establishments^11^.

The classification was performed in accordance with the guidelines of the Dietary Guidelines for the Brazilian Population^12^ and the 2017–2018 Pesquisa de Orçamentos Familiares (POF – Family Budget Survey)^2^, with adaptations that allowed for greater detail in the intermediate categories. It should be noted that, to identify the food purchasing profile in commercial establishments, purchase records from the Collective Purchase Logbook and the Individual Purchase Questionnaire of the 2017–2018 POF were considered.

Thus, according to the food purchase profile, each establishment was classified into five categories: fresh, mixed fresh, mixed processed, ultra-processed, and other mixed (Chart). This distinction was based on the estimated proportion of items belonging to different food groups, considering the purchasing profile by state, based on data from the 2017–2018 POF.

**Chart:** New proposal for classifying RAIS establishments based on the percentage of food purchases.

Establishment profile	Criteria
Fresh	Establishments with 50% or more of food purchases of fresh or minimally processed foods
Mixed fresh^a^	Establishments with 40% or more of food purchases consisting of fresh or minimally processed foods and processed foods, and less than 20% of purchases consisting of ultra-processed foods
Mixed processed^a^	Households with 40% or more of their food purchases consisting of ultra-processed foods and less than 20% of fresh or minimally processed foods; or households with at least 70% of their food purchases consisting of ultra-processed and/or processed foods and less than 20% of fresh or minimally processed foods
Ultra-processed	Households with 50% or more of purchases consisting of ultra-processed foods
Other mixed	Other, unclassified

RAIS: Relação Anual de Informações Sociais (Annual Report on Social Information).

^a^ Not classified in the previous study by CAISAN (2018).

The food group dictionary used in the classification was developed based on documents from CAISAN (2018), which were derived from the Dietary Guidelines for the Brazilian Population and the NOVA classification, encompassing the categories of fresh or minimally processed foods, processed foods, ultra-processed foods, culinary ingredients, and culinary preparations. Unclassified items, such as alcoholic beverages, followed the criteria established in CAISAN (2018), and new products from the 2017–2018 POF were classified based on the similarity between descriptions and the authors’ judgment.

The dictionary was applied to products recorded in the Collective Purchasing Logbook and the Individual Purchasing Questionnaire, with the correspondence between the instruments based on the similarity of food descriptions. Based on this procedure, the food procurement profile for each type of establishment was estimated, allowing for its classification into the proposed categories. Thus, the same type of establishment may have a different classification in different states.

Subsequently, the establishments were grouped into two categories: (1) healthy: establishments classified as fresh, mixed fresh, and other mixed, in addition to the public food service facilities considered in this study (open-air markets, popular restaurants, markets, produce markets, and public grocery stores); (2) unhealthy: establishments classified as mixed processed and ultra-processed.

It is noteworthy that public food service facilities were included as healthy establishments only in the intramunicipal analysis (91 municipalities with more than 300,000 inhabitants), thereby expanding the diversity of access points analyzed. The restriction to larger municipalities is due to the greater complexity of the urban network, higher density and diversity of establishments, and better availability of geocoded data, allowing for a more robust application of high-resolution accessibility analyses.

The sources for SAN facilities were MapaSAN 2022, the Consumer Protection Institute’s market database, kitchens registered on the Ministry of Development and Social Assistance, Family and Fight Against Hunger, municipal government websites, and interviews with some managers from the priority municipalities of the Estratégia Alimenta Cidades (Food for Cities Strategy). The Estratégia Alimenta Cidades is a federal government initiative aimed at strengthening urban food systems and expanding access to adequate and healthy food.

For all 5,570 Brazilian municipalities, the availability of healthy and unhealthy establishments was assessed based on the density of establishments per 10,000 inhabitants. To characterize inequalities in distribution, we used the 25th percentile (P25) as a reference for the lowest density of healthy establishments and the 75th percentile (P75) as a reference for the highest density of unhealthy establishments.

This nationwide analysis informed the subsequent steps, conducted at the intramunicipal level, which included assessing population access, mapping food deserts and food swamps, and examining associations with socioeconomic indicators in the 91 municipalities with the largest populations.

### Estimation of Population Access to Food Establishments

The delineation of urbanized areas utilized the grid from the Instituto Brasileiro de Geografia e Estatística (IBGE - Brazilian Institute of Geography and Statistics)^13^ and the 111 million addresses from the Cadastro Nacional de Endereços para Fins Estatísticos (National Address Registry for Statistical Purposes). Hexagons were chosen as the territorial units of analysis due to their topological properties, which minimize the Modifiable Areal Unit Problem (MAUP) through their neighborhood and connectivity components, while also providing flexibility to aggregate units at different scales.

Only hexagons with ≥ 20 households were considered, totaling 145,730 units of analysis. The population was redistributed based on the 2022 Census, proportionally to household density. The road network was constructed using OpenStreetMap (Geofabrik), and routes were calculated using the r5r package (R) for walking (3.6 km/h), adjusted for topography (SRTM 30 m).

Accessibility was measured by the number of establishments that could be reached within a 15-minute walk, normalized per 1,000 inhabitants, distinguishing between locations classified as healthy and unhealthy.

### Definition and Mapping of Food Deserts and Food Swamps

In this study, the identification of food deserts and food swamps was based on a cumulative accessibility metric, defined as the number of establishments reachable on foot within 15 minutes from each H3 hexagon (≈ 0.1 km^2^), normalized per 1,000 inhabitants. Based on this metric, the territories were classified as: food deserts, when located in the first quartile of accessibility to healthy establishments (≤ 5 per 1,000 inhabitants); and food swamps, when located in the last quartile of accessibility to unhealthy establishments (> 15 per 1,000 inhabitants).

**Figure 3:**
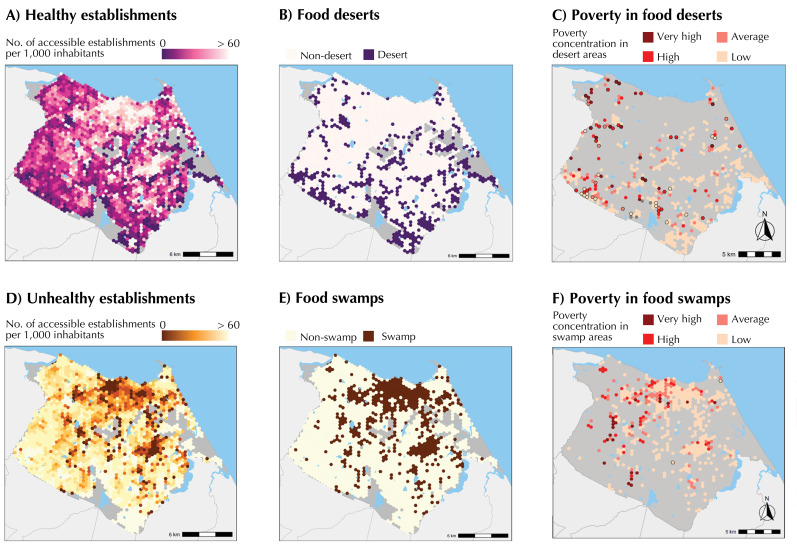
Food deserts and food swamps in Fortaleza, Ceará.

Note: number of (a) healthy and (d) unhealthy establishments. Spatial distribution of (b) food deserts and (e) food swamps in Fortaleza, Ceará. Concentration of poverty areas (very high indicating high concentration of poverty) in areas of (c) food deserts and (f) food swamps in Fortaleza, Ceará.

The analysis focused on 91 Brazilian municipalities with more than 300,000 inhabitants, which together account for approximately 77 million inhabitants. At this scale, food deserts were defined by low relative accessibility to healthy food establishments, while food swamps reflected high relative accessibility to unhealthy food establishments. Intra-urban zoning used H3 hexagons, allowing for the identification of vulnerable sectors and the generation of detailed maps highlighting patterns of limited access to food, as demonstrated in Fortaleza in Figure 3.

It should be noted that the concepts of food deserts and food swamps adopted in this study are operationalized relatively, based on the distribution of accessibility within the set of municipalities analyzed. Thus, these categories do not represent an absolute absence or absolute excess of establishments, but rather comparative conditions of lesser or greater access within the Brazilian urban context. This approach allows for the identification of internal spatial inequalities, even though it does not establish universal thresholds for adequate food access.

### Integration of Food Deserts and Food Wetlands with Socioeconomic Indicators

In the final stage, food deserts and food swamps were cross-referenced with socioeconomic and demographic indicators from the IBGE Census, including *per capita* income, educational attainment, poverty, and social vulnerability. This procedure aimed to assess the relationship between socioeconomic conditions and the spatial distribution of the food environment. Case studies were also conducted in selected cities, such as Fortaleza, to detail the overlap of these environments with areas of greater vulnerability, allowing for the identification of priority territories for intervention through public policies.

### Data Analysis

The descriptive analysis included the calculation of absolute and relative frequencies, as well as measures of central tendency and dispersion, to characterize the distribution of establishments. For the national-scale analysis, the densities of healthy and unhealthy establishments were described by country, region, state, and municipal population size. At the intramunicipal level (91 municipalities with more than 300,000 inhabitants), results regarding food deserts and food swamps were presented for Brazil and by region; stratification by state was not performed due to the small number of municipalities in some states.

The choropleth maps were created using ArcGIS Pro 3.3 (Esri) software, while statistical analyses were conducted using R software, version 4.3.2^14^.

### Ethical Considerations

All procedures followed protocols for reproducibility and standardization of geographic and statistical variables. As this study was based exclusively on secondary, publicly available databases and did not involve individual identification of participants, there was no need for submission to a Research Ethics Committee, in accordance with Resolution No. 510/2016 of the National Health Council.

## RESULTS

### National Distribution of the Density of Healthy and Unhealthy Establishments

**Figure 2:**
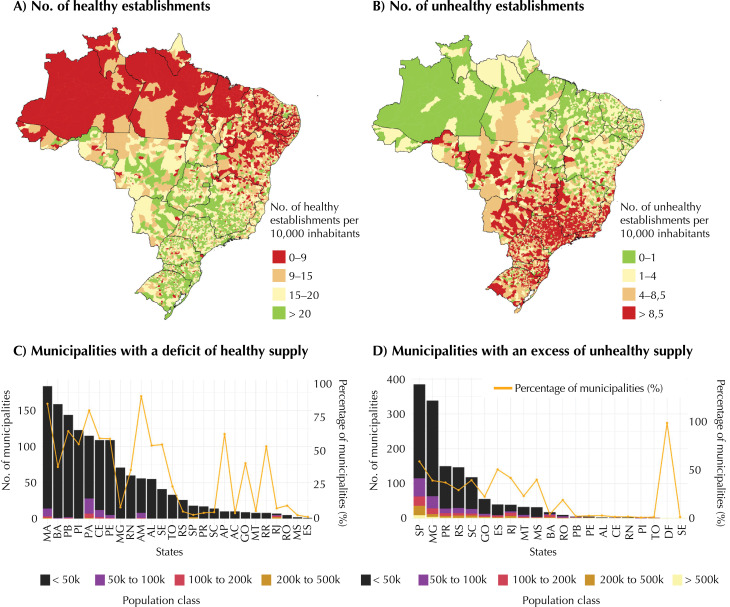
Distribution of the density of healthy and unhealthy establishments in Brazilian municipalities.

Note: Density of establishments predominantly (a) healthy (majority of fresh foods) and (b) unhealthy (predominance of ultra-processed), standardized per 10,000 inhabitants. Number of municipalities by state, classified by population size, with predominantly (c) healthy and (d) unhealthy establishments.

The density of healthy establishments was higher in the South, Southeast, and parts of the Central-West, especially in municipalities with larger populations. In contrast, low values prevailed in the North and Northeast, below 9 per 10,000 inhabitants (Figure 2A).

For unhealthy establishments, the density was lower in the North and Northeast, generally below 1 per 10,000 inhabitants, and higher in the South and Southeast, exceeding 8.5 in some locations (Figure 2B). The lower availability of healthy food outlets was concentrated in small municipalities, particularly in Maranhão, Bahia, Pará, and Piauí, while the excess of unhealthy food outlets was most pronounced in São Paulo, Minas Gerais, Paraná, and Rio Grande do Sul (Figures 2C–D).

### Food Deserts and Food Swamps and Social Inequalities

Approximately 25 million Brazilians reside in areas classified as food deserts, representing 32.3% of the total population of the 91 municipalities with more than 300,000 inhabitants. The proportion of the population residing in food deserts showed significant regional variation. The highest percentages were observed in the North (40.0%), Northeast (34.0%), and Southeast (37.0%) regions, while the lowest were in the South and Central-West, both at 23.0%. In absolute numbers, the Southeast region has the largest population living in food deserts, approximately 15 million people (Table 1).

**Table 1:** Estimates of the absolute and relative numbers of the total populationa and the population registered in the Cadastro Único with a *per capita* family income below half the minimum wage (low-income and living in poverty) in the 91 Brazilian municipalities with more than 300,000 inhabitants residing in food deserts, by macro-region.

Region	Total population in food deserts	Percentage (%) of the population in food deserts	Low-income and impoverished population in food deserts	Percentage (%) of the low-income and impoverished population in food deserts
North	2,694,284	44.6%	933,674	44.7%
Northeast	4,697,893	31.0%	1,612,708	33.3%
Central-West	1,616,605	24.4%	296,504	25.1%
Southeast	14,615,272	34.6%	3,635,603	42.6%
South	1,473,251	19.2%	244,636	23.1%
**Brazil**	**25,097,305**	**32.3%**	**6,723,125**	**38.0%**

^a^ Population in urbanized areas.

Approximately 15 million Brazilians live in food swamps, which corresponds to 19% of the total population of these municipalities. The presence of food swamps was highest in the South (28.2%), followed by the Central-West (25.0%). In absolute numbers, the Southeast region has the largest population living in food swamps, approximately 8.8 million people (Table 2).

**Table 2:** Estimates of the absolute and relative numbers of the total populationa and the population registered in the Cadastro Único with a *per capita* family income below half the minimum wage (low-income and living in poverty) in the 91 Brazilian municipalities with more than 300,000 inhabitants residing in food-producing wetlands, by macro-region.

Region	Total population in food wetlands	Percentage (%) of the population in food-producing wetlands	Low-income and impoverished population in food-producing wetlands	Percentage (%) of the low-income and impoverished population in food-producing wetlands
North	470,276	7.8%	125,187	6.0%
Northeast	1,668,763	11.0%	315,738	6.5%
Central-West	1,657,793	25.0%	219,769	18.6%
Southeast	8,808,357	20.9%	999,161	11.7%
South	2,163,040	28.2%	178,495	16.9%
**Brazil**	**14,768,229**	**19.0%**	**1,838,350**	**10.4%**

^a^ Population in urbanized areas.

Considering social vulnerability data, approximately 6.7 million low-income people living in poverty reside in food deserts, which corresponds to 38% of the total population registered in the Cadastro Único with a monthly *per capita* family income below half the minimum wage residing in these cities. In relative terms, the North region has the highest proportion (44.7%) and the South region the lowest (23.1%) (Table 1).

Approximately 1.8 million low-income and impoverished people live in food swamps, representing 10.4% of the total population in the Cadastro Único with a monthly *per capita* family income below half the minimum wage residing in these cities. In relative terms, the Central-West region has the highest proportion (18.6%), followed by the South (16.9%), while the North (6%) and Northeast (6.5%) regions have the lowest proportions (Table 2).

To illustrate the geographic distribution of food deserts and food wetlands in large urban centers, the results for Fortaleza (CE) are presented in the figures (Figure 3). The city has central areas with more than 60 healthy food establishments per 1,000 inhabitants, in contrast to outlying areas characterized by low availability (Figure 3A). Food deserts (Figure 3B) are concentrated mainly in regions farther from the center, while food swamps (Figure 3E) are distributed more diffusely, with higher density in specific commercial corridors. In these same regions, poverty is high (3C and 3F), revealing the overlap between a lack of healthy foods, a predominance of ultra-processed foods, and high socioeconomic vulnerability.

## DISCUSSION

The results of this study confirm the existence of territorial inequalities in access to fresh or minimally processed foods in Brazil. An unequal distribution of healthy and unhealthy establishments was identified across regions and population sizes, with a notable higher concentration of municipalities with lower densities of healthy establishments in the North and Northeast regions and in municipalities with smaller populations (< 50,000 inhabitants). Among municipalities with more than 300,000 inhabitants, it was observed that one-third and one-fifth of the population reside, respectively, in food deserts and food swamps. The percentage of people living in food deserts was higher in the North and lower in the South, while the percentage of people residing in food swamps was higher in the South and Central-West and lower in the North and Northeast. Furthermore, the overlap with socioeconomic indicators revealed that individuals with greater social vulnerability are disproportionately concentrated in these territories, particularly noting the high percentage of low-income people and those living in poverty who reside in food desert areas, reinforcing the structural link between poverty and food exclusion.

The observed regional differences should be interpreted in light of the structural specificities of the Brazilian territory. In the North and Northeast regions, the greater presence of food deserts may reflect not only socioeconomic inequalities but also lower urban density, greater territorial dispersion, and logistical limitations in food distribution. On the other hand, in the South and Southeast regions, the higher density of unhealthy establishments may be associated with more consolidated consumer markets, greater urbanization, and a greater presence of retail and fast-food chains. These patterns indicate that food deserts and food swamps are not opposing phenomena, but distinct expressions of regional food systems, shaped by specific economic, urban, and commercial dynamics.

These findings are consistent with national and international studies highlighting inequality in access to healthy foods^15^. The mapping conducted in 2018^11^ had already identified food deserts in several regions of Brazil, although based on more limited methodologies and without integration with social vulnerability data. This study indicated that, in 15 of the 21 Brazilian state capitals analyzed, the group of subdistricts with the lowest presence of establishments offering healthy foods also corresponded to the group of subdistricts with the lowest income, and that, in most capitals, there is a direct relationship between increased income and increased density of healthy food retail outlets^11^.

Other studies have also highlighted territorial inequalities in access to healthy foods^18^. An ecological study conducted in Porto Alegre (RS) found that areas at high risk of health vulnerability, with higher percentages of black and indigenous people, illiterate individuals, and people living on less than the minimum wage, were about twice as likely to be classified as food deserts^19^. Similarly, another study conducted in Recife (PE) found that census tracts classified as food deserts exhibit greater social vulnerability, poorer income conditions, and limited access to essential services, in addition to concentrating higher numbers of illiterate, black, mixed-race, and indigenous people^20^. In that same study, food swamps prevailed in census tracts with lower vulnerability, where the population is predominantly white, literate, higher-income, and with better sanitation conditions^20^. Honório et al.^21^ assessed the evolution of food deserts and food swamps in the Belo Horizonte Metropolitan Region between 2008 and 2020. The study showed that the proportion of census tracts classified as food deserts decreased during the period, while that of food swamps increased. Notably, food swamps showed a marked increase precisely in the most vulnerable census tracts.

National and international studies show that living in urban areas classified as food deserts or food swamps has been associated with poorer dietary outcomes and a higher risk of obesity and chronic noncommunicable diseases^22, 23^.

This study builds upon the mapping conducted in 2018^11^. One of the main innovations involves improving the accuracy of the geolocation of food retail establishments by identifying complete addresses. Another innovation was the development and application of a new methodology for mapping food deserts and food swamps, incorporating physical access to private food retail establishments and providing an intramunicipal-scale analysis for the 91 municipalities with more than 300,000 inhabitants, among which are the priority municipalities of the first implementation cycle of the Alimenta Cidades Strategy.

The use of a cumulative accessibility metric based on walking time, normalized by population and operationalized using an H3 hexagonal grid, provides a more realistic reflection of the daily experience of Brazil’s urban population. Overlaying this data with household income data from the Cadastro Único allows for the identification of priority areas for food security actions and policies. Furthermore, incorporating public food security facilities broadens the understanding of food supply beyond the private sector.

These methodological advances have made it possible not only to map availability but also to identify, with greater precision, critical zones of overlap between food exclusion and socioeconomic vulnerability. In this way, they contribute strategic information for prioritizing public food security policies and for improving the efficiency of local food systems from a healthy, sustainable, and inclusive perspective.

Some limitations must be acknowledged. The analysis was based on RAIS^10^ data, which cover only the formal sector, excluding informal establishments that play an important role in many localities. Furthermore, the study focused on urbanized areas, excluding rural areas, where dynamics of food access differ substantially, which limits the generalizability of the results to these contexts. The operational definition of food deserts and food swamps was based on quartiles, which allows for relative comparisons but does not establish absolute parameters of food adequacy. Factors such as prices, food quality, or cultural preferences, which decisively influence actual consumption, were also not considered. Finally, this is a cross-sectional study, which does not allow for capturing the temporal dynamics of food environments.

Despite the limitations noted, the study makes progress on several methodological and analytical fronts. Notable features include the use of temporal accessibility metrics, the precision of geolocation, the integration of multiple socioeconomic databases, and the inclusion of public FNS facilities. These advances yield robust and comparable evidence capable of informing public policies, improving the monitoring of food environments, and supporting territorially precise management of FNS-related instruments in Brazil.

The findings have direct implications for territorial planning and the formulation of public food and nutrition security policies. First, they indicate the need for local policies that offer tax and credit incentives to encourage the establishment of businesses selling fresh food in areas classified as food deserts. Second, they reinforce the strategic role of urban and peri-urban agriculture, the food supply network, and public food and nutrition security facilities (EqSAN), such as open-air markets and affordable restaurants, in mitigating inequalities in access.

Furthermore, the results support the adoption of regulatory measures to restrict the sale and advertising of ultra-processed foods in vulnerable areas, as well as the integration of the food environment into urban planning, transportation, and health policies. In this regard, the role of the public sector is reinforced as the primary agent in transforming the local food environment into a healthier space, contributing to the implementation of public food and nutrition security policies and ensuring the human right to adequate and healthy food.

Such actions are consistent with national commitments, such as the Plano Brasil Sem Fome (Brazil Without Hunger Plan)^2^⁰ and the III Plano Nacional de Segurança Alimentar e Nutricional (3rd National Food and Nutrition Security Plan) and can contribute to promoting territorial equity in access to healthy food. In this sense, the identification of food deserts and food swamps should be understood as an analytical tool to guide public policies, rather than as a rigid normative categorization of territories. The complexity of food environments requires contextualized approaches that simultaneously consider physical access, socioeconomic conditions, cultural patterns, and local supply dynamics.

## Data Availability

Data is available upon request to the corresponding author.
