# Supplementary material for: Food environment in Brazil: food deserts and food swamps in large urban cities
Source: Rev Saude Publica. 2026 Jul 17;60:e30. doi: 10.11606/s1518-8787.2026060007379 (PMC13379222; doi:10.11606/s1518-8787.2026060007379)
Supplement: Translation

# Translation: Ambiente alimentar no Brasil: desertos e pântanos em grandes cidades urbanas

**Authors:** Giovani William Gianetti, Mariana Giannotti, Gabriel Caldeira, Naila Takahashi, Cláudia Roberta Bocca Santos, Ariene Silva do Carmo, Rodrigo Fernando Maule, Gisele Ane Bortolini, Ronaldo Torres, Rogério de S Nóia-Júnior, Bruna Pitasi Arguelhes, Patrícia Chaves Gentil, Lilian Rahal dos Santos, Márcia Muchagata, Sergio Paganini Martins

**Affiliations:** I Universidade de São Paulo. Instituto para Governança Territorial e Políticas Públicas. Piracicaba, SP, Brasil; II Universidade de São Paulo. Escola Politécnica. São Paulo, SP, Brasil; III Centro de Estudos da Metrópole. São Paulo, SP, Brasil; IV Ministério do Desenvolvimento e Assistência Social, Família e Combate à Fome. Secretaria Nacional de Segurança Alimentar e Nutricional. Brasília, DF, Brasil; V Universidade Federal de Ouro Preto. Departamento de Nutrição Clínica e Social. Ouro Preto, MG, Brasil

**Keywords:** Ambiente Alimentar, Políticas Públicas, Segurança Alimentar, Saúde Pública

## Abstract

**OBJETIVO:**

Avaliar a disponibilidade de estabelecimentos saudáveis e não saudáveis no Brasil e, com isso, mapear desertos e pântanos alimentares. Analisar os mapas de desertos e pântanos de forma integrada a indicadores de desigualdade social.

**MÉTODOS:**

Dados da Relação Anual de Informações Sociais (RAIS, 2022), integrados aos perfis de consumo da Pesquisa de Orçamentos Familiares (POF, 2017–2018), foram utilizados para classificar os estabelecimentos segundo o Guia Alimentar para a População Brasileira. Em escala municipal, estimou-se a densidade de estabelecimentos com perfil saudável e não saudável para os municípios brasileiros. Em escala intramunicipal, foram identificados desertos e pântanos em municípios urbanos com mais de 300 mil habitantes, cruzando os resultados com indicadores de renda do Cadastro Único.

**RESULTADOS:**

Em nível municipal, observou-se que as regiões Norte e Nordeste concentram a maioria dos municípios com baixa densidade de estabelecimentos saudáveis, enquanto Sul e Sudeste apresentam maiores densidades de estabelecimentos não saudáveis. Em escala intramunicipal, cerca de 25 milhões de pessoas residem em desertos alimentares, o que corresponde a 32,3% da população dos 91 municípios analisados e, aproximadamente, 15 milhões vivem em pântanos alimentares (19% da população). Entre os indivíduos do Cadastro Único com renda *per capita* inferior a meio salário-mínimo, 38% vivem em desertos alimentares e 10% em pântanos.

**CONCLUSÃO:**

Os resultados evidenciam a sobreposição entre vulnerabilidade socioeconômica e ambientes alimentares adversos, reforçando a urgência de políticas públicas voltadas à regulação do ambiente alimentar, ao fortalecimento de equipamentos públicos de abastecimento e à promoção da equidade territorial no acesso a alimentos saudáveis.

## INTRODUÇÃO

O Brasil vivencia rápidas transformações sociais, econômicas e territoriais, marcadas pelo avanço da urbanização, pela intensificação de eventos climáticos extremos e pela persistência de desigualdades estruturais que limitam o acesso a uma alimentação adequada e saudável^1^. Em 2022, a insegurança alimentar grave atingia 33 milhões de pessoas, ou 15% da população, 27 milhões delas em áreas urbanas^2^. Em 2023, esse número caiu para 8,7 milhões de pessoas, das quais mais de 7 milhões viviam em cidades, refletindo avanços em políticas de proteção social^3^. Já em 2024, 6,48 milhões de brasileiros estavam em situação de fome. Apesar disso, formas múltiplas de má nutrição persistem, incluindo deficiências de micronutrientes, excesso de peso e obesidade. Entre 2006 e 2023, a prevalência de excesso de peso em adultos subiu de 42,6% para 61,4%, e a obesidade mais que dobrou, alcançando 24,3%^4^.

Esse cenário expressa uma transição alimentar acelerada, marcada pela substituição de alimentos *in natura* ou minimamente processados por ultraprocessados, sobretudo entre grupos socialmente vulnerabilizados. Estimativas indicam que esses produtos já respondem por 19,7% da ingestão calórica nacional, com crescimento mais acentuado em áreas rurais, entre populações negras e indígenas, de menor escolaridade e renda, e nas regiões Norte e Nordeste^1^. Essa dinâmica é sustentada por sistemas alimentares hegemônicos, organizados pela lógica da industrialização e da padronização, em detrimento de práticas locais e sustentáveis. Tais sistemas são reconhecidos como motores da chamada sindemia global, caracterizada pela convergência de desnutrição, obesidade e mudanças climáticas, que compartilham determinantes comuns e se reforçam mutuamente, ampliando riscos para populações em maior situação de vulnerabilidade^5^.

Um dos componentes centrais desses sistemas é o ambiente alimentar, definido como o conjunto de fatores físicos, econômicos, políticos e socioculturais que moldam escolhas alimentares e influenciam diretamente padrões de consumo e saúde^8^. Nesse contexto, os conceitos de desertos e pântanos alimentares têm sido amplamente empregados. Desertos alimentares correspondem a áreas com disponibilidade e acessibilidade restritas a alimentos saudáveis, enquanto pântanos alimentares se caracterizam pela predominância de estabelecimentos que comercializam, sobretudo, alimentos ultraprocessados^9^. Ambos configuram expressões espaciais da desigualdade no acesso a dietas adequadas, associando-se a piores indicadores de saúde^10^.

O mapeamento desses ambientes tem importância estratégica para orientar políticas públicas de segurança alimentar e nutricional (SAN). Em 2018, a Câmara Interministerial de Segurança Alimentar e Nutricional (Caisan) realizou o primeiro levantamento nacional de desertos alimentares. Mais recentemente, a atualização desse mapeamento foi assumida como compromisso pelo Ministério do Desenvolvimento e Assistência Social, Família e Combate à Fome, ao apresentar uma nova estratégia voltada ao ambiente alimentar urbano.

Diante desse contexto, o presente estudo tem como objetivo avaliar a disponibilidade de estabelecimentos saudáveis e não saudáveis em todos os municípios brasileiros e mapear a presença de desertos e pântanos alimentares em municípios com mais de 300 mil habitantes, integrando dados de desigualdade social. O estudo busca oferecer uma visão abrangente e comparável do ambiente alimentar urbano, subsidiando estratégias regulatórias e de planejamento territorial que ampliem o acesso equitativo a alimentos saudáveis.

## MÉTODOS

Trata-se de um estudo de delineamento ecológico, que contemplou a caracterização da densidade de estabelecimentos comerciais de alimentos no âmbito nacional (n = 5.570), tendo como unidade de análise os municípios brasileiros; e o mapeamento de desertos e pântanos alimentares em escala intramunicipal para todos os municípios brasileiros com mais de 300 mil habitantes (n = 91), tendo como unidade de análise os hexágonos dentro de cada município.

O estudo foi estruturado em quatro etapas integradas (Figura 1):

**Figura 1:** Fluxo metodológico para a construção da tipologia e mapeamento dos ambientes alimentares no Brasil.

- Etapa 1 – Identificação, classificação e zoneamento dos estabelecimentos comerciais de alimentos.
- Etapa 2 – Caracterização do ambiente alimentar em escala nacional: foram analisados os 5.570 municípios brasileiros para caracterizar a disponibilidade de estabelecimentos alimentícios classificados como saudáveis (predomínio de alimentos *in natura* ou minimamente processados) e não saudáveis (predomínio de ultraprocessados). A métrica utilizada foi a densidade de estabelecimentos por 10 mil habitantes. Para destacar as desigualdades na distribuição, consideraram-se o 25º percentil (P25) da densidade de estabelecimentos saudáveis e o 75º percentil (P75) da densidade de estabelecimentos não saudáveis.
- Etapas 3 a 5 – Análise em escala intramunicipal. A análise concentrou-se nos 91 municípios brasileiros com população superior a 300 mil habitantes: na Etapa 3, estimou-se o acesso populacional a alimentos *in natura* por métricas de densidade e distribuição espacial; na Etapa 4, foram mapeados os desertos alimentares (escassez de estabelecimentos saudáveis) e os pântanos alimentares (predomínio de estabelecimentos não saudáveis) e, na Etapa 5, os resultados foram associados a indicadores socioeconômicos, revelando a sobreposição entre desigualdades sociais e restrições ao acesso a alimentos saudáveis (Figura 1).

### Identificação, Classificação e Zoneamento dos Estabelecimentos

Os estabelecimentos varejistas de alimentos foram identificados a partir da base da Relação Anual de Informações Sociais (RAIS), referente ao ano de 2022^10^, utilizando os 18 códigos da Classificação Nacional de Atividades Econômicas que abrangem os estabelecimentos de aquisição de alimentos^11^.

A classificação foi realizada conforme a proposta do Guia Alimentar para a População Brasileira^12^ e da Pesquisa de Orçamentos Familiares (POF) 2017–2018^2^, com adaptações que permitiram maior detalhamento das categorias intermediárias. Destaca-se que, com o objetivo de identificar o perfil de aquisição de alimentos nos estabelecimentos comerciais, foram considerados os registros de aquisição da Caderneta de Aquisição Coletiva e do Questionário de Aquisição Individual da POF 2017–2018.

Assim, conforme o perfil de aquisição de alimentos, cada estabelecimento foi classificado em cinco categorias: *in natura*, misto *in natura*, misto processado, ultraprocessado e outros mistos (Quadro). Essa distinção se baseou na proporção estimada de itens pertencentes aos diferentes grupos alimentares, considerando o perfil de aquisição por unidade federativa, com base nos dados da POF 2017–2018.

**Quadro:** Nova proposta de classificação dos estabelecimentos da RAIS com base no percentual de aquisição de alimentos.

Perfil do estabelecimento	Critérios
*In natura*	Estabelecimentos com 50% ou mais das aquisições de alimentos *in natura* ou minimamente processados
Misto *in natura* ^a^	Estabelecimentos com 40% ou mais das aquisições de alimentos *in natura* ou minimamente processados e processados e menos de 20% de aquisições de ultraprocessados
Misto processado^a^	Estabelecimentos com 40% ou mais das aquisições de ultraprocessados e menos de 20% de alimentos *in natura* ou minimamente processados*;* ou estabelecimentos com pelo menos 70% das aquisições de alimentos ultraprocessados e/ou processados e menos de 20% *in natura* ou minimamente processados
Ultraprocessado	Estabelecimentos com 50% ou mais das aquisições de alimentos ultraprocessados
Outros mistos^a^	Demais não classificados

RAIS: Relação Anual de Informações Sociais.

^a^ Não classificados no estudo anterior da Caisan (2018).

O dicionário de grupos de alimentos utilizado na classificação foi construído com base nos documentos da Caisan (2018), elaborado a partir do Guia Alimentar para a População Brasileira e da classificação NOVA, contemplando as categorias de alimentos *in natura* ou minimamente processados, processados, ultraprocessados, ingredientes culinários e preparações culinárias. Itens não classificados, como bebidas alcoólicas, seguiram os critérios estabelecidos em Caisan (2018), e novos produtos da POF 2017–2018 foram classificados com base na similaridade entre descrições e no julgamento dos autores.

O dicionário foi aplicado aos produtos registrados na Caderneta de Aquisição Coletiva e no Questionário de Aquisição Individual, sendo a correspondência entre os instrumentos realizada com base na similaridade das descrições dos alimentos. A partir desse procedimento, estimou-se o perfil de aquisição de alimentos para cada tipo de estabelecimento, permitindo sua classificação nas categorias propostas. Dessa forma, um mesmo tipo de estabelecimento pode ter classificação distinta em diferentes estados.

Posteriormente, os estabelecimentos foram agrupados em duas categorias: (1) saudáveis: estabelecimentos classificados como *in natura*, misto *in natura* e outros mistos, além dos equipamentos públicos de SAN considerados neste estudo (feiras livres, restaurantes populares, mercados, sacolões e quitandas públicas); (2) não saudáveis: estabelecimentos classificados como misto processado e ultraprocessado.

Destaca-se que os equipamentos de SAN foram incluídos como estabelecimentos saudáveis apenas na análise intramunicipal (91 municípios com mais de 300 mil habitantes), ampliando a diversidade de pontos de acesso analisados. A restrição aos municípios de maior porte deve-se à maior complexidade da rede urbana, maior densidade e diversidade de estabelecimentos e melhor disponibilidade de dados geocodificados, permitindo a aplicação mais robusta das análises de acessibilidade em alta resolução.

As fontes dos equipamentos de SAN foram o MapaSAN 2022, a base de feiras do Instituto de Defesa do Consumidor, as cozinhas cadastradas no Ministério do Desenvolvimento e Assistência Social, Família e Combate à Fome (MDS), os portais das prefeituras municipais e entrevistas com alguns gestores dos municípios prioritários da Estratégia Alimenta Cidades. A Estratégia Alimenta Cidades é uma iniciativa do governo federal voltada ao fortalecimento dos sistemas alimentares urbanos e à ampliação do acesso à alimentação adequada e saudável.

Para todos os 5.570 municípios brasileiros, avaliou-se a disponibilidade de estabelecimentos saudáveis e não saudáveis por meio da densidade de estabelecimentos a cada 10 mil habitantes. Para caracterizar desigualdades na distribuição, utilizamos o 25º percentil (P25) como referência de menor densidade de estabelecimentos saudáveis e o 75º percentil (P75) como referência de maior densidade de estabelecimentos não saudáveis.

Essa análise em escala nacional fundamentou as etapas seguintes, realizadas em escala intramunicipal, que incluíram a avaliação do acesso populacional, o mapeamento de desertos e pântanos alimentares e a associação com indicadores socioeconômicos nos 91 municípios de maior tamanho populacional.

### Estimativa do Acesso da População aos Estabelecimentos Alimentares

A delimitação das áreas urbanizadas utilizou a malha do Instituto Brasileiro de Geografia e Estatística (IBGE)^13^ e os 111 milhões de endereços do Cadastro Nacional de Endereços para Fins Estatísticos. Optou-se pelos hexágonos como unidades territoriais de análise, devido às suas propriedades topológicas, que minimizam o problema da unidade de área modificável (ou MAUP, do inglês *Modifiable Areal Unit Problem*), por sua componente de vizinhança e conectividade, além de conferirem flexibilidade para agregar as unidades em diferentes escalas.

Foram considerados apenas hexágonos com ≥ 20 domicílios, totalizando 145.730 unidades de análise. A população foi redistribuída com base no Censo 2022, proporcionalmente à densidade de domicílios. A rede viária foi construída a partir do *OpenStreetMap* (Geofabrik), e as rotas foram calculadas no pacote r5r (R) para deslocamento a pé (3,6 km/h), ajustadas pela topografia (SRTM 30 m).

A acessibilidade foi mensurada pelo número de estabelecimentos passíveis de serem alcançados em até 15 minutos de deslocamento a pé, normalizado por 1.000 habitantes, distinguindo-se entre pontos classificados como saudáveis e não saudáveis.

### Definição e Mapeamento de Desertos e Pântanos Alimentares

Neste estudo, a identificação de desertos e pântanos alimentares baseou-se em uma métrica de acessibilidade cumulativa, definida como o número de estabelecimentos alcançáveis a pé em até 15 minutos a partir de cada hexágono H3 (≈ 0,1 km^2^), normalizada por 1.000 habitantes. Com base nessa métrica, os territórios foram classificados em: desertos alimentares, quando situados no primeiro quartil de acessibilidade a estabelecimentos saudáveis (≤ 5 por 1.000 hab.); e pântanos alimentares, quando situados no último quartil de acessibilidade a estabelecimentos não saudáveis (> 15 por 1.000 hab.).

A análise concentrou-se em 91 municípios brasileiros com mais de 300 mil habitantes, que reúnem aproximadamente 77 milhões de habitantes. Nessa escala, os desertos foram definidos pela baixa acessibilidade relativa a estabelecimentos saudáveis, enquanto os pântanos refletiram alta acessibilidade relativa a estabelecimentos não saudáveis. O zoneamento intraurbano utilizou hexágonos H3, permitindo identificar setores vulneráveis e gerar mapas detalhados que evidenciam padrões de acesso limitado a alimentos, como demonstrado em Fortaleza na Figura 3.

**Figura 3:** Desertos e pântanos alimentares em Fortaleza, Ceará.

Nota: número de estabelecimentos (a) saudáveis e (d) não saudáveis. Distribuição espacial de (b) desertos e (e) pântanos alimentares em Fortaleza, Ceará. Concentração de áreas de pobreza (muito alta indicando alta concentração de pobreza) em áreas de (c) desertos e (f) pântanos alimentares em Fortaleza, Ceará.

Ressalta-se que os conceitos de desertos e pântanos alimentares adotados neste estudo são operacionalizados de forma relativa, com base na distribuição da acessibilidade dentro do conjunto de municípios analisados. Assim, essas categorias não representam uma ausência absoluta ou excesso absoluto de estabelecimentos, mas sim condições comparativas de menor ou maior acesso no contexto urbano brasileiro. Essa abordagem permite identificar desigualdades espaciais internas, ainda que não estabeleça limiares universais de adequação alimentar.

### Integração de Desertos e Pântanos Alimentares com Indicadores Socioeconômicos

Na etapa final, os desertos e os pântanos alimentares foram cruzados com indicadores socioeconômicos e demográficos do Censo do IBGE, incluindo renda *per capita*, escolaridade, pobreza e vulnerabilidade social. Esse procedimento teve como objetivo avaliar a relação entre as condições socioeconômicas e a distribuição espacial do ambiente alimentar. Também foram realizados estudos de caso em cidades selecionadas, como Fortaleza, para detalhar a sobreposição desses ambientes com áreas de maior vulnerabilidade, permitindo identificar territórios prioritários para intervenção por meio de políticas públicas.

### Análise de Dados

A análise descritiva contemplou o cálculo de frequências absolutas e relativas, bem como medidas de posição e dispersão, para caracterizar a distribuição dos estabelecimentos. Para a análise em escala nacional, as densidades de estabelecimentos saudáveis e não saudáveis foram descritas por país, região, unidade federativa e tamanho populacional dos municípios. Na escala intramunicipal (91 municípios com mais de 300 mil habitantes), os resultados referentes a desertos e pântanos alimentares foram apresentados para o Brasil e por regiões, não sendo realizada a estratificação por unidade federativa devido ao número reduzido de municípios em alguns estados.

Os mapas coropléticos foram elaborados no *software* ArcGIS Pro 3.3 (Esri), enquanto as análises estatísticas foram conduzidas no *software* R, versão 4.3.2^14^.

### Aspectos Éticos

Todos os procedimentos seguiram protocolos de reprodutibilidade e padronização das variáveis geográficas e estatísticas. Por se tratar de um estudo baseado exclusivamente em bases de dados secundárias, de domínio público e sem identificação individual dos participantes, não houve necessidade de submissão a Comitê de Ética em Pesquisa, conforme a Resolução nº 510/2016 do Conselho Nacional de Saúde.

## RESULTADOS

### Distribuição Nacional da Densidade de Estabelecimentos Saudáveis e Não Saudáveis

A densidade de estabelecimentos saudáveis foi maior nas regiões Sul, Sudeste e em parte do Centro-Oeste, especialmente em municípios com maior população. Já nas regiões Norte e Nordeste prevaleceram valores baixos, inferiores a 9 por 10 mil habitantes (Figura 2A).

**Figura 2:** Distribuição da densidade de estabelecimentos saudáveis e não saudáveis nos municípios brasileiros.

Nota: densidade de estabelecimentos predominantemente (a) saudáveis (oferta majoritária de alimentos *in natura*) e (b) não saudáveis (predominância de ultraprocessados), padronizada por 10 mil habitantes. Número de municípios por estado da federação, e classificados por tamanho populacional, com estabelecimentos predominantemente (c) saudáveis e (d) não saudáveis.

Para os estabelecimentos não saudáveis, a densidade foi menor no Norte e no Nordeste, geralmente abaixo de 1 por 10 mil habitantes, e mais alta no Sul e no Sudeste, superando 8,5 em alguns locais (Figura 2B). A menor disponibilidade de estabelecimentos de aquisição de alimentos saudáveis concentrou-se em municípios pequenos, sobretudo no Maranhão, na Bahia, no Pará e no Piauí, enquanto o excesso de estabelecimentos não saudáveis destacou-se em São Paulo, Minas Gerais, Paraná e Rio Grande do Sul (Figuras 2C–D).

### Desertos e Pântanos Alimentares e Desigualdades Sociais

Cerca de 25 milhões de brasileiros residem em áreas classificadas como desertos alimentares, o que representa 32,3% da população total dos 91 municípios com mais de 300 mil habitantes. A proporção da população residente em desertos alimentares apresentou variação regional expressiva. As maiores porcentagens foram observadas nas regiões Norte (40,0%), Nordeste (34,0%) e Sudeste (37,0%), enquanto as menores ocorreram no Sul e Centro-Oeste, ambos com 23,0%. Em números absolutos, a região Sudeste concentra a maior população residente em desertos alimentares, cerca de 15 milhões de pessoas (Tabela 1).

**Tabela 1:** Estimativa do número absoluto e relativo da população totala e da população inscrita no Cadastro Único com renda *per capita* familiar inferior a meio salário-mínimo (baixa renda e em situação de pobreza) nos 91 municípios brasileiros com mais de 300 mil habitantes que residem em desertos alimentares, segundo macrorregião.

Região	População total em desertos alimentares	Percentual (%) da população em desertos alimentares	População de baixa renda e em situação de pobreza em desertos alimentares	Percentual (%) da população de baixa renda e em situação de pobreza em desertos alimentares
Norte	2.694.284	44,6%	933.674	44,7%
Nordeste	4.697.893	31,0%	1.612.708	33,3%
Centro-Oeste	1.616.605	24,4%	296.504	25,1%
Sudeste	14.615.272	34,6%	3.635.603	42,6%
Sul	1.473.251	19,2%	244.636	23,1%
**Brasil**	**25.097.305**	**32,3%**	**6.723.125**	**38,0%**

^a^ População em área urbanizada.

Aproximadamente 15 milhões de brasileiros vivem em pântanos alimentares, o que corresponde a 19% da população total desses municípios. A presença de pântanos alimentares foi maior na região Sul (28,2%), seguida da Centro-Oeste (25,0%). Em números absolutos, a região Sudeste concentra a maior população residente em pântanos alimentares, cerca de 8,8 milhões de pessoas (Tabela 2).

**Tabela 2:** Estimativa do número absoluto e relativo da população totala e da população inscrita no Cadastro Único com renda *per capita* familiar inferior a meio salário-mínimo (baixa renda e em situação de pobreza) nos 91 municípios brasileiros com mais de 300 mil habitantes que residem em pântanos alimentares, segundo macrorregião.

Região	População total em pântanos alimentares	Percentual (%) da população em pântanos alimentares	População de baixa renda e em situação de pobreza em pântanos alimentares	Percentual (%) da população de baixa renda e em situação de pobreza em pântanos alimentares
Norte	470.276	7,8%	125.187	6,0%
Nordeste	1.668.763	11,0%	315.738	6,5%
Centro-Oeste	1.657.793	25,0%	219.769	18,6%
Sudeste	8.808.357	20,9%	999.161	11,7%
Sul	2.163.040	28,2%	178.495	16,9%
**Brasil**	**14.768.229**	**19,0%**	**1.838.350**	**10,4%**

^a^ População em área urbanizada.

Considerando os dados de vulnerabilidade social, cerca de 6,7 milhões de pessoas de baixa renda e em situação de pobreza vivem em desertos alimentares, o que corresponde a 38% da população total inscrita no Cadastro Único com renda *per capita* familiar mensal inferior a meio salário-mínimo residente nessas cidades. Em termos relativos, a região Norte apresenta a maior proporção (44,7%) e a região Sul a menor (23,1%) (Tabela 1).

Aproximadamente 1,8 milhão de pessoas de baixa renda e em situação de pobreza vivem em pântanos alimentares, o que representa 10,4% da população total do Cadastro Único com renda *per capita* familiar mensal inferior a meio salário-mínimo residente nessas cidades. Em termos relativos, a região Centro-Oeste apresenta a maior proporção (18,6%), seguida da região Sul (16,9%), enquanto as regiões Norte (6%) e Nordeste (6,5%) apresentam as menores proporções (Tabela 2).

Para exemplificar a distribuição geográfica de desertos e pântanos alimentares em grandes centros urbanos, apresentam-se, em imagens, os resultados para Fortaleza (CE) (Figura 3). A cidade apresenta áreas centrais com mais de 60 estabelecimentos saudáveis por 1.000 habitantes, contrastando com zonas periféricas caracterizadas por baixa disponibilidade (Figura 3A). Os desertos alimentares (Figura 3B) concentram-se principalmente em regiões mais afastadas do centro, enquanto os pântanos alimentares (Figura 3E) distribuem-se de forma mais difusa, com maior densidade em corredores comerciais específicos. Nessas mesmas regiões, a pobreza é elevada (Figuras 3C e 3F), revelando a sobreposição entre falta de alimentos saudáveis, predominância de ultraprocessados e alta vulnerabilidade socioeconômica.

## DISCUSSÃO

Os resultados deste estudo confirmam a existência de desigualdades territoriais no acesso a alimentos *in natura* ou minimamente processados no Brasil. Identificou-se uma distribuição desigual de estabelecimentos saudáveis e não saudáveis entre regiões e tamanhos populacionais, com destaque para a maior concentração de municípios com menor densidade de estabelecimentos saudáveis nas regiões Norte e Nordeste e em municípios de menor tamanho populacional (< 50 mil habitantes). Entre os municípios com mais de 300 mil habitantes, observou-se que um terço e um quinto da população residem, respectivamente, em áreas de desertos e de pântanos alimentares. O percentual de pessoas que vivem em desertos alimentares foi maior na região Norte e menor na região Sul, enquanto o percentual de pessoas residentes em pântanos alimentares foi maior nas regiões Sul e Centro-Oeste e menor nas regiões Norte e Nordeste. Além disso, a sobreposição com indicadores socioeconômicos revelou que indivíduos em maior vulnerabilidade social se concentram de forma desproporcional nesses territórios, com destaque para o elevado percentual de pessoas de baixa renda e em situação de pobreza que residem em áreas de desertos alimentares, reforçando a ligação estrutural entre pobreza e exclusão alimentar.

As diferenças regionais observadas devem ser interpretadas à luz das especificidades estruturais do território brasileiro. Nas regiões Norte e Nordeste, a maior presença de desertos alimentares pode refletir não apenas desigualdades socioeconômicas, mas também menor densidade urbana, maior dispersão territorial e limitações logísticas na distribuição de alimentos. Por outro lado, nas regiões Sul e Sudeste, a maior densidade de estabelecimentos não saudáveis pode estar associada a mercados consumidores mais consolidados, maior urbanização e maior presença de redes varejistas e de alimentação rápida. Esses padrões indicam que desertos e pântanos alimentares não são fenômenos opostos, mas expressões distintas de sistemas alimentares regionais, moldados por dinâmicas econômicas, urbanas e comerciais específicas.

Esses achados estão em consonância com estudos nacionais e internacionais que evidenciam a desigualdade no acesso a alimentos saudáveis^15^. O mapeamento conduzido em 2018^11^ já havia identificado desertos alimentares em várias regiões do Brasil, embora com base em metodologias mais restritas e sem integração com dados de vulnerabilidade social. Esse estudo apontou que, em 15 das 21 capitais brasileiras analisadas, o grupo de subdistritos com menor presença de estabelecimentos que oferecem alimentos saudáveis correspondia também ao grupo de subdistritos de menor renda, e que, na maioria das capitais, existe uma relação direta entre o aumento da renda e o aumento da densidade de pontos de comercialização de alimentos saudáveis^11^.

Outros estudos também evidenciaram desigualdades territoriais no acesso a alimentos saudáveis^18^. Um estudo ecológico realizado em Porto Alegre (RS) identificou que áreas com alto risco de vulnerabilidade sanitária, com maior percentual de pessoas negras e indígenas, de analfabetos e de indivíduos que vivem com menos de um salário-mínimo, eram cerca de duas vezes mais propensas a serem classificadas como desertos alimentares^19^. De forma semelhante, outro estudo realizado em Recife (PE) identificou que os setores censitários considerados desertos alimentares apresentam maior vulnerabilidade social, piores condições de renda e de acesso a serviços essenciais, além de concentrarem maior número de pessoas analfabetas, pretas, pardas e indígenas^20^. Nesse mesmo estudo, os pântanos alimentares prevaleceram nos setores censitários de menor vulnerabilidade, onde predomina a população branca, alfabetizada, de maior renda e com melhores condições de saneamento^20^. Honório et al.^21^ avaliaram a evolução dos desertos e pântanos alimentares na Região Metropolitana de Belo Horizonte entre 2008 e 2020. O estudo mostrou que a taxa de setores censitários classificados como desertos alimentares diminuiu no período, enquanto a de pântanos alimentares aumentou. Destaca-se que os pântanos alimentares apresentaram aumento acentuado justamente nos setores censitários de maior vulnerabilidade.

Estudos nacionais e internacionais mostram que viver em áreas urbanas classificadas como desertos ou pântanos alimentares tem sido associado a piores desfechos dietéticos e a um risco maior de obesidade e doenças crônicas não transmissíveis^22, 23^.

Este estudo avança em relação ao mapeamento realizado em 2018^11^. Uma das principais inovações envolve a melhoria na precisão da geolocalização dos estabelecimentos comerciais de alimentos, por meio da identificação de endereços completos. Outra inovação foi a construção e aplicação de uma nova metodologia para o mapeamento de desertos e pântanos alimentares, incorporando o acesso físico aos estabelecimentos comerciais privados de alimentos e proporcionando uma análise em escala intramunicipal para os 91 municípios com mais de 300 mil habitantes, entre os quais se encontram os municípios prioritários do primeiro ciclo de implementação da Estratégia Alimenta Cidades.

A utilização de uma métrica de acessibilidade cumulativa em tempo de deslocamento a pé, normalizada pela população e operacionalizada em malha hexagonal H3, aproxima-se de forma mais realista da experiência cotidiana da população urbana brasileira. A sobreposição com os dados de renda familiar do Cadastro Único permite identificar áreas prioritárias para ações e políticas de SAN. Além disso, a incorporação dos equipamentos públicos de segurança alimentar amplia a compreensão da oferta alimentar para além do setor privado.

Esses avanços metodológicos permitiram não apenas mapear a disponibilidade, mas também identificar, com maior precisão, zonas críticas de sobreposição entre a exclusão alimentar e a vulnerabilidade socioeconômica. Dessa forma, contribuem com informações estratégicas para a priorização de políticas públicas de SAN e para o aprimoramento da eficiência dos sistemas alimentares locais, a partir de uma perspectiva saudável, sustentável e inclusiva.

Algumas limitações devem ser reconhecidas. A análise baseou-se em dados da RAIS^10^, que abrangem apenas o setor formal, excluindo estabelecimentos informais que desempenham papel importante em diversas localidades. Além disso, o estudo concentrou-se em áreas urbanizadas, não contemplando as áreas rurais, cujas dinâmicas de acesso aos alimentos diferem substancialmente, o que limita a generalização dos resultados para esses contextos. A definição operacional de desertos e pântanos baseou-se em quartis, o que permite comparações relativas, mas não estabelece parâmetros absolutos de adequação alimentar. Também não foram considerados fatores como preços, qualidade dos alimentos ou preferências culturais, que influenciam decisivamente o consumo efetivo. Por fim, trata-se de um recorte transversal, que não permite capturar a dinâmica temporal dos ambientes alimentares.

Apesar das limitações indicadas, o estudo avança em diversas frentes metodológicas e analíticas. Destacam-se o uso de métricas de acessibilidade temporal, a precisão da geolocalização, a integração de múltiplas bases de dados socioeconômicos e a inclusão de equipamentos públicos de SAN. Esses avanços resultam em evidências robustas e comparáveis, capazes de subsidiar políticas públicas, aprimorar o monitoramento dos ambientes alimentares e apoiar uma gestão territorialmente precisa dos instrumentos voltados à SAN no Brasil.

Os achados têm implicações diretas para o planejamento territorial e para a formulação de políticas públicas de SAN. Em primeiro lugar, indicam a necessidade de políticas locais que ofereçam incentivos fiscais e de crédito para estimular a instalação de estabelecimentos que comercializem alimentos *in natura* em áreas classificadas como desertos alimentares. Em segundo lugar, reforçam o papel estratégico da agricultura urbana e periurbana, da rede de abastecimento alimentar e dos equipamentos públicos de segurança alimentar e nutricional (EqSAN), como feiras livres e restaurantes populares, na mitigação das desigualdades de acesso.

Além disso, os resultados apoiam a adoção de medidas regulatórias para restringir a aquisição e a publicidade de ultraprocessados em territórios vulnerabilizados, bem como a integração do ambiente alimentar às políticas de planejamento urbano, de transporte e de saúde. Nesse sentido, reforça-se o papel do setor público como principal agente na transformação do ambiente alimentar local em um espaço mais saudável, colaborando para a implementação de políticas públicas de SAN e garantindo o direito humano à alimentação adequada e saudável.

Tais ações são coerentes com compromissos nacionais, como o Plano Brasil Sem Fome^2^⁰ e o III Plano Nacional de Segurança Alimentar e Nutricional, e podem contribuir para a promoção da equidade territorial no acesso à alimentação saudável. Nesse sentido, a identificação de desertos e pântanos alimentares deve ser compreendida como uma ferramenta analítica para orientar políticas públicas, e não como uma categorização normativa rígida dos territórios. A complexidade dos ambientes alimentares requer abordagens contextualizadas, que considerem simultaneamente o acesso físico, as condições socioeconômicas, os padrões culturais e as dinâmicas locais de abastecimento.

## Data Availability

Os dados estão disponíveis mediante solicitação ao autor correspondente.
